# Acute stroke alert activation, emergency service use, and reperfusion therapy in Sweden

**DOI:** 10.1002/brb3.654

**Published:** 2017-03-15

**Authors:** Marie Eriksson, Eva‐Lotta Glader, Bo Norrving, Birgitta Stegmayr, Kjell Asplund

**Affiliations:** ^1^ Department of StatisticsUSBE, Umeå UniversityUmeåSweden; ^2^ Department of Public Health and Clinical MedicineUmeå UniversityUmeåSweden; ^3^ Section of NeurologyDepartment of Clinical SciencesLund UniversityLundSweden

**Keywords:** ambulatory care, prenotification, reperfusion, stroke, stroke alert, thrombolysis, time‐to‐treatment

## Abstract

**Objectives:**

Ambulance services and stroke alerts reduce the time from stroke onset to acute stroke diagnosis. We describe the use of stroke alerts and ambulance services in different hospitals and patient groups and their relationship with reperfusion therapy.

**Methods:**

This nationwide study included 49,907 patients admitted with acute stroke who were registered in The Swedish Stroke Register (Riksstroke) in 2011–2012.

**Results:**

The proportions of patients admitted as stroke alerts out of all acute stroke admissions varied from 12.2% to 45.7% in university hospitals (*n* = 9), 0.5% to 38.7% in specialized nonuniversity hospitals (*n* = 22), and 4.2% to 40.3% in community hospitals (*n* = 41). Younger age, atrial fibrillation (AF), living in an institution, reduced consciousness upon admission, and hemorrhagic stroke were factors associated with a higher probability of stroke alerts. Living alone, primary school education, non‐European origin, previous stroke, diabetes, smoking, and dependency in activities of daily living (ADL) were associated with a lower probability of stroke alert. The proportion of patients arriving at the hospital by ambulance varied from 60.3% to 94.5%. Older age, living alone, primary school education, being born in a European country, previous stroke, AF, dependency in ADL, living in an institution, reduced consciousness upon admission, and hemorrhagic stroke were associated with ambulance services. Hospital stroke alert frequencies correlated strongly with reperfusion rates (*r *=* *.75).

**Conclusion:**

Acute stroke alerts have a significant potential to improve stroke reperfusion rates. Prehospital stroke management varies conspicuously between hospitals and patient groups, and the elderly and patients living alone have a markedly reduced likelihood of stroke alerts.

## 1 Introduction

High‐priority ambulance service and stroke prenotification are key components in reducing prehospital delay and time to diagnosis in patients with suspected acute stroke (Fassbender et al., 2013; Meretoja et al., 2012, 2013; Oostema, Nasiri, Chassee, & Reeves, 2014). Stroke alerts (prenotification by ambulance staff members or initiated in the emergency department) have been shown to be strongly associated with shorter in‐hospital delay to the onset of treatment (door‐to‐needle time [DNT]) and higher proportions of patients being treated with thrombolysis (Binning et al., 2014; Lin et al., 2012a; McKinney et al., 2013; Patel, Rose, O'Brien, & Rosamond, 2011; Prabhakaran, O'Neill, Stein‐Spencer, Walter, & Alberts, 2013; Ragoschke‐Schumm et al., 2014). Additional benefits of prenotification have been reported in patients with warfarin‐associated intracerebral hemorrhage, in whom the time to reversal of anticoagulation was shortened (Dowlatshahi et al., 2013). Rapid triage and immediate stroke team activation (stroke alert) are important components of Target: Stroke, which is a nationwide quality‐improvement initiative of the American Heart Association and the American Stroke Association to improve the care of stroke in the US (Fonarow et al., 2011, 2014), and they are recommended in the guidelines issued by the European Stroke Organisation (2008).

In Sweden, national guidelines include stroke alerts as one of the indicators of high‐quality acute stroke care (National Board of Health and Welfare, 2015). However, annual reports from the Swedish Stroke Register (Riksstroke, 2014) show marked regional and between‐hospital variations in the use of ambulance services and stroke alerts (Riksstroke) (as has also been reported in the US; Lin et al., 2012b). DNT also vary substantially between hospitals (Stecksen, Glader, Asplund, Norrving, & Eriksson, 2014).

We aimed to describe (1) between‐hospital variations in the use of stroke alerts and ambulance services in different types of hospitals; (2) longitudinal changes in stroke alerts and the use of stroke alerts and ambulance services in different patient groups; and (3) time from onset to hospital admission and the association between stroke alert frequency, ambulance services, and reperfusion therapy rates.

## 2 Material and Methods

### 2.1 Material

Riksstroke was established in 1994 to monitor, support, and improve the quality of stroke care in Sweden. All hospitals admitting acute stroke patients in Sweden participate (nine university hospitals, 22 specialized nonuniversity hospitals, and 41 community hospitals in 2012), and Riksstroke has an estimated coverage of 94% of all acute stroke patients treated in Swedish hospitals (Riksstroke). Riksstroke includes information on living conditions and comorbidities prior to stroke, acute stroke treatment, and secondary prevention, and patient‐reported outcome is followed up after 3 and 12 months. Details on what information is collected are available at the Riksstroke website http://www.riksstroke.org/eng/.

The main analysis in this study included all 49,907 stroke events (ICD 10‐codes: I61, I63, or I64) in patients ≥18 years that were registered in Riksstroke in 2011–2012. The descriptive analysis of time trends in stroke alert frequencies included 200,133 stroke events registered from 2005 to 2012.

### 2.2 Variable definitions

All Swedish hospitals have stroke alert protocols to quickly and accurately identify and triage stroke patients. SOS Alarm, the service coordinating ambulance calls in Sweden, is classifying stroke as a level 1 priority, irrespective of the time from onset (SOS Alarm, 2016). In Riksstroke, stroke alerts include prehospital notifications from ambulance and alerts at the emergency unit for patients presenting with stroke symptoms. Stroke alerts and whether the patient arrived in an ambulance are registered in Riksstroke as “yes”, “no”, or “unknown”. Onset to admission time (OAT) was categorized as ≤3, 3–4.5, 4.5–24, >24 hr, or unknown. Reperfusion therapy was defined as thrombolysis, thrombectomy, or both.

Level of consciousness upon admission to the hospital was used as a proxy for stroke severity and was registered using three levels based on the Reaction Level Scale (RLS; Starmark, Stalhammar, & Holmgren, 1988). Alert corresponded to RLS 1, drowsy to RLS 2–3, and unconscious to RLS 4–8. Independence in activities of daily living (ADL) was defined as the patient being able to manage dressing, using the toilet, and walking unassisted.

Information on patient education and country of birth were retrieved through individual linkage with the Longitudinal Integration Database for Health Insurance and Labor Market Studies (LISA by its Swedish acronym; Statistics Sweden, 2016), which is managed by Statistics Sweden, using personal identification numbers. Highest achieved education was grouped into primary school, secondary school, or university education. Country of birth was grouped into Sweden, other Nordic countries (Finland, Norway, Denmark, and Iceland), other European countries, and outside Europe.

Hospitals were categorized into university, specialized nonuniversity, or community hospitals based on their degree of specialization (Asplund, Sukhova, Wester, Stegmayr, & Riksstroke, 2015).

### 2.3 Statistical methods

Unadjusted proportions are presented with 95% confidence intervals for subgroups of patients. The Mantel–Haenszel test was used to test if stroke alert frequency increased over time. We used multiple logistic regression to simultaneously analyze the association between the independent factors of sex, age group, socioeconomic status (education, country of birth, and living alone), comorbidities (previous stroke, atrial fibrillation [AF], diabetes, treatment for high blood pressure, and dependency in ADL), institutional living, smoking status, level of consciousness at admission, stroke subtype, and outcome (probability of stroke alert, ambulance transport to hospital, and OAT time <3 hr). To adjust for individual hospital variation (e.g., caused by population and geographical differences, or variation in stroke alert criteria), hospital was included and modeled as a fixed effect (Varewyck, Goetghebeur, Eriksson, & Vansteelandt, 2014). The effect of hospital type was analyzed in a separate model not including individual hospital effects. Pearson correlation was used to assess the association between hospital stroke alert frequency, ambulance service, and hospital reperfusion rate. We performed a separate analysis of in‐hospital stroke alerts in the subgroup of patients who did not use ambulance.

Education level and smoking status were missing for >2% of the patients, and these were included in the analysis by adding a separate category (“unknown”) in the multiple regression. Patients with other missing variables were omitted from the multiple regression analysis. A difference with a *p*‐value <.05 was considered statistically significant. We used SAS 9.4 for statistical analyses.

## 3 Results

### 3.1 Stroke alerts

The stroke alert frequency increased from 4.6% in 2005 to 23.1% in 2012 (from 8.9% to 29.3% in university hospitals, from 3.9% to 24.0% in specialized nonuniversity hospitals, and from 3.6% to 19.8% in community hospitals, all *p* < .001). In the two most recent years, 2011 and 2012, the frequency was 27.5% (hospital range: 12.2%–45.7%) in university hospitals, 21.7% (range: 0.5%–38.7%) in specialized nonuniversity hospitals, and 18.3% (range: 4.2%–40.3%) in community hospitals. Compared with community hospitals, stroke alert frequencies were higher in specialized nonuniversity hospitals (OR 1.27, 95% CI: 1.20–1.34) and in university hospitals (OR 1.58, 95% CI: 1.49–1.69) after adjustment for patient‐level factors (Table 1).

**Table 1:** Stroke alert frequency (%) with 95% confidence intervals (95% CI), 2011–2012

Variable	Category	Valid *N*	Prop. (95% CI)	Adj. OR (95% CI)
Sex	Women	23,411	20.0 (19.4–20.5)	0.99 (0.94–1.04)
	Men	24,955	23.3 (22.8–23.9)	Ref.
Age group	18–54	2,945	28.8 (27.2–30.5)	Ref.
	55–64	5,448	25.8 (24.7–27.0)	0.94 (0.85–1.05)
	65–74	11,255	25.5 (24.7–26.3)	0.92 (0.83–1.01)
	75–84	15,724	21.6 (20.9–22.2)	0.78 (0.71–0.87)
	85+	12,994	15.3 (14.6–15.9)	0.52 (0.47–0.58)
Education	Unknown	1,343	25.2 (22.8–27.5)	1.03 (0.86–1.24)
	Primary	22,384	19.1 (18.6–19.6)	Ref.
	Secondary	17,166	23.2 (22.6–23.9)	1.08 (1.02–1.14)
	University	7,473	25.3 (24.4–26.3)	1.01 (0.94–1.08)
Country of birth	Missing	402	35.8 (31.1–40.5)	–
	Sweden	41,980	21.4 (21.0–21.8)	Ref.
	Other Nordic	2,767	21.9 (20.4–23.5)	1.00 (0.91–1.11)
	Other Europe	2,141	24.6 (22.7–26.4)	1.02 (0.91–1.14)
	Other	1,076	23.0 (20.5–25.6)	0.79 (0.68–0.93)
Living alone	Missing	257	17.9 (13.2–22.6)	–
	No	23,938	26.5 (26.0–27.1)	Ref.
	Yes	24,171	17.0 (16.5–17.5)	0.63 (0.60–0.66)
Previous stroke	Missing	306	14.7 (10.7–18.7)	–
	No	36,222	22.4 (22.0–22.9)	Ref.
	Yes	11,838	19.6 (18.9–20.3)	0.90 (0.85–0.96)
Atrial fibrillation	Missing	300	23.3 (18.5–28.1)	–
	No	34,294	21.4 (21.0–21.9)	Ref.
	Yes	13,772	22.3 (21.7–23.0)	1.25 (1.19–1.32)
Diabetes	Missing	163	25.8 (19.0–32.6)	–
	No	38,244	22.3 (21.9–22.7)	Ref.
	Yes	9,959	19.4 (18.6–20.2)	0.85 (0.80–0.90)
Hypertensive medication	Missing	314	24.8 (20.0–29.6)	–
	No	18,623	23.3 (22.6–23.9)	Ref.
	Yes	29,429	20.7 (20.2–21.2)	0.96 (0.92–1.01)
Smoker	Unknown	3,783	22.6 (21.3–24.0)	1.01 (0.92–1.11)
	No	38,450	21.6 (21.2–22.0)	Ref.
	Yes	6,133	21.9 (20.8–22.9)	0.87 (0.81–0.94)
ADL‐dependent	Missing	978	15.8 (13.6–18.1)	–
	No	41,630	22.8 (22.4–23.2)	Ref
	Yes	5,758	14.9 (14.0–15.8)	0.70 (0.63–0.77)
Living in an institution	Missing	151	19.2 (12.9–25.6)	–
	No	43,424	22.3 (21.9–22.7)	Ref.
	Yes	4,791	16.6 (15.6–17.7)	1.13 (1.01–1.25)
Level of consciousness	Missing	551	13.6 (10.7–16.5)	–
	Alert	39,436	21.0 (20.6–21.4)	Ref.
	Drowsy	5,934	27.8 (26.7–29.0)	1.70 (1.59–1.83)
	Unconscious	2,445	20.1 (18.5–21.7)	1.02 (0.90–1.15)
Stroke subtype	Hemorrhagic	5,672	27.2 (26.0–28.3)	1.25 (1.17–1.35)
	Ischemic	42,012	21.2 (20.8–21.5)	Ref.
	Unspecified	682	10.6 (8.2–12.9)	0.57 (0.44–0.74)
Hospital type	University	9,893	27.5 (26.6–28.4)	1.58 (1.49–1.69)
	Specialized nonuniversity	19,242	21.7 (21.1–22.2)	1.27 (1.20–1.34)
	Community	16,692	18.3 (17.7–18.8)	Ref.

Adjusted odds ratios (adj. OR) with 95% CI from the multiple logistic regression model including all variables in the table.

**Figure 1:**
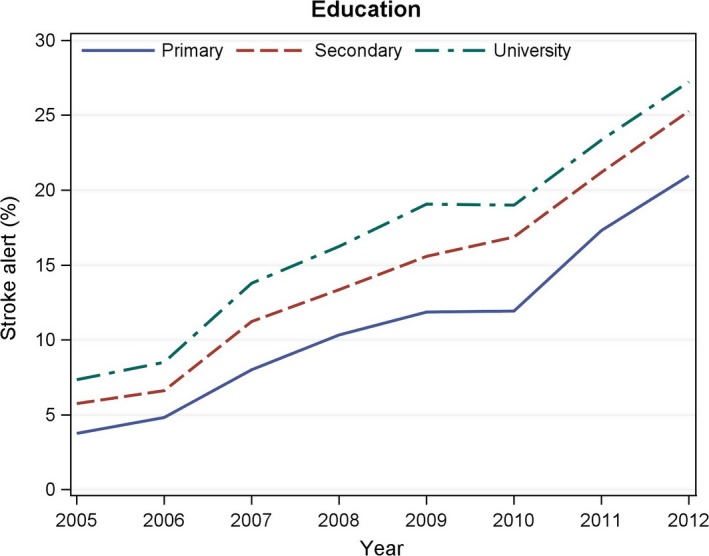
Education and longitudinal changes in stroke alert frequencies 2005–2012

After adjustment for other patient factors, the probability of stroke alert was lower in patients older than 74 years than in younger patients. Living alone, primary school education, non‐European origin, previous stroke, diabetes, smoking, and dependency in ADL were other factors associated with a lower probability of stroke alert in the multiple regression model. The probability of stroke alert was higher in patients with AF, those who were living in an institution, those who were drowsy at hospital admission, and those who had a hemorrhagic stroke (Table 1). The observed socioeconomic differences in stroke alert frequencies persisted throughout the study period (Figure 1).

The frequency of in‐hospital stroke alerts was 6.7% in the 11,372 patients who did not use ambulance transport to hospital 2011–2012. After adjustment for other factors, older patients, patients born outside the Nordic countries, and patients living alone had lower probability of in‐hospital stroke alerts (Table S1). In‐hospital stroke alert frequencies were higher in university hospitals than in community hospitals.

### 3.2 Ambulance services

The proportion of patients arriving at the hospital by ambulance in 2011–2012 was 74.3% (hospital range: 65.9%–90.9%) in university hospitals, 72.8% (range: 66.9%–80.3%) in specialized nonuniversity hospitals, and 72.7% (range: 60.3%–94.5%) in community hospitals.

Older age, living alone, primary school education, European origin, previous stroke, AF, dependency in ADL, living in an institution, being drowsy or unconscious at admission, and hemorrhagic stroke were factors associated with use of ambulance services in the multiple regression (Table 2). Patients arriving at the hospital by ambulance also had a higher probability of stroke alert (29.6% vs. 6.7%, *p* < .001).

**Table 2:** Ambulance transport to hospital (%) with 95% confidence intervals (95% CI), 2011–2012

Variable	Category	Valid *N*	Prop. (95% CI)	Adj. OR (95% CI)
Sex	Women	21,073	76.1 (75.5–76.7)	1.00 (0.96–1.06)
	Men	22,422	71.3 (70.7–71.9)	Ref.
Age group	18–54	2,619	60.9 (59.0–62.7)	Ref.
	55–64	4,891	61.7 (60.3–63.0)	1.04 (0.93–1.16)
	65–74	10,089	67.4 (66.5–68.3)	1.28 (1.16–1.41)
	75–84	14,139	75.3 (74.6–76.0)	1.64 (1.49–1.82)
	85+	11,757	84.7 (84.0–85.3)	2.34 (2.09–2.61)
Education	Unknown	1,210	76.9 (74.6–79.3)	1.09 (0.90–1.32)
	Primary	19,938	77.2 (76.6–77.8)	Ref.
	Secondary	15,531	71.0 (70.3–71.7)	0.91 (0.86–0.96)
	University	6,816	68.5 (67.4–69.6)	0.85 (0.79–0.91)
Country of birth	Missing	362	70.2 (65.4–74.9)	–
	Sweden	37,747	74.0 (73.5–74.4)	Ref.
	Other Nordic	2,479	73.7 (71.9–75.4)	0.99 (0.90–1.10)
	Other Europe	1,912	71.4 (69.4–73.5)	0.92 (0.82–1.03)
	Other	995	65.4 (62.5–68.4)	0.74 (0.64–0.87)
Living alone	Missing	214	65.4 (59.0–71.8)	–
	No	21,472	69.1 (68.5–69.7)	Ref.
	Yes	21,809	78.1 (77.6–78.7)	1.12 (1.07–1.18)
Previous stroke	Missing	266	77.4 (72.4–82.5)	–
	No	32,497	71.4 (71.0–71.9)	Ref.
	Yes	10,732	80.1 (79.3–80.8)	1.32 (1.24–1.40)
Atrial fibrillation	Missing	278	82.0 (77.5–86.6)	–
	No	30,705	70.5 (70.0–71.0)	Ref.
	Yes	12,512	81.0 (80.3–81.7)	1.33 (1.26–1.41)
Diabetes	Missing	149	80.5 (74.1–87.0)	–
	No	34,397	73.7 (73.2–74.1)	Ref.
	Yes	8,949	73.2 (72.3–74.1)	0.99 (0.94–1.05)
Hypertensive medication	Missing	301	80.1 (75.5–84.6)	–
	No	16,731	72.0 (71.3–72.7)	Ref.
	Yes	26,463	74.6 (74.0–75.1)	0.97 (0.92–1.02)
Smoker	Unknown	3,318	80.8 (79.5–82.1)	1.10 (0.99–1.23)
	No	34,697	74.0 (73.5–74.4)	Ref.
	Yes	5,480	66.9 (65.6–68.1)	1.03 (0.96–1.10)
ADL‐dependent	Missing	862	89.6 (87.5–91.6)	–
	No	37,395	71.0 (70.6–71.5)	Ref
	Yes	5,238	89.3 (88.5–90.1)	1.73 (1.56–1.94)
Living in an institution	Missing	129	81.4 (74.6–88.2)	–
	No	38,958	71.6 (71.2–72.1)	Ref.
	Yes	4,408	90.9 (90.1–91.8)	1.69 (1.07–1.18)
Level of consciousness	Missing	551	13.6 (10.7–16.5)	–
	Alert	39,436	21.0 (20.6–21.4)	Ref.
	Drowsy	5,934	27.8 (26.7–29.0)	4.06 (3.64–4.54)
	Unconscious	2,445	20.1 (18.5–21.7)	4.68 (3.88–5.65)
Stroke subtype	Hemorrhagic	5,672	27.2 (26.0–28.3)	1.68 (1.54–1.83)
	Ischemic	42,012	21.2 (20.8–21.5)	Ref.
	Unspecified	682	10.6 (8.2–12.9)	0.69 (0.55–0.86)
Hospital type	University	8,846	74.3 (73.4–75.2)	1.12 (1.05–1.20)
	Specialized nonuniversity	17,823	72.8 (72.1–73.4)	1.10 (1.04 1.16)
	Community	14,956	72.7 (72.0–73.4)	Ref.

Adjusted odds ratios (Adj. OR) with 95% CI from multiple logistic regression model including all variables in table.

### 3.3 Onset to admission time

In total, 15,904 (31.9%) of the patients were admitted to a hospital within 3 hr of the onset of symptoms, 3,838 (7.7%) within 3–4.5 hr, 14,062 (28.2%) within 4.5–24 hr, and 7,747 (15.5%) later than 24 hr from onset. OAT was unknown for 8,356 (16.7%) of the patients.

In the multiple logistic regression, adjusting for other patient factors, an OAT within 3 hr was associated with younger age, secondary or university education, being married or cohabiting, having AF, not having diabetes, nonsmoking, living in an institution, being independent in ADL, being drowsy or unconscious on admission, and suffering from hemorrhagic stroke (Table 3).

**Table 3:** Onset to admission time (OAT) 2011–2012

Variable	Category	Onset to admission time (OAT)					Adj. OR for OAT <3 hr (95% CI)
		Unknown (%)	<3 hr (%)	3–4.5 hr (%)	4.5–24 hr (%)	>24 hr (%)	
Sex	Women	17.8	30.6	8.1	29.0	14.5	1.01 (0.97–1.06)
	Men	15.8	33.1	7.3	27.4	16.5	Ref.
Age group	18–54	15.4	36.2	6.6	22.9	19.0	Ref.
	55–64	14.0	32.4	6.9	24.0	19.6	0.87 (0.79–0.96)
	65–74	14.9	32.8	6.8	27.5	18.0	0.85 (0.77–0.92)
	75–84	17.3	32.5	7.7	27.8	14.7	0.83 (0.76–0.91)
	85+	19.1	29.1	9.0	31.0	11.8	0.74 (0.68–0.82)
Education	Unknown	18.6	32.0	7.1	26.9	15.3	0.94 (0.80–1.10)
	Primary	17.9	30.7	7.9	28.8	14.6	Ref
	Secondary	16.1	32.3	7.3	27.8	16.5	1.05 (1.01–1.10)
	University	14.4	34.2	8.0	27.3	16.1	1.07 (1.01–1.13)
Country of birth	Sweden	16.7	32.0	7.8	28.3	15.3	Ref.
	Other Nordic	17.3	30.3	6.9	29.0	16.5	0.98 (0.90–1.07)
	Other Europe	18.4	30.5	7.0	26.8	17.4	0.96 (0.87–1.06)
	Other	16.1	30.6	7.0	26.1	20.2	0.93 (0.81–1.07)
Living alone	No	13.7	37.4	7.3	26.1	15.6	Ref.
	Yes	19.6	26.4	8.1	30.3	15.5	0.59 (0.56–0.62)
Previous stroke	No	16.4	31.8	7.5	27.8	16.5	Ref.
	Yes	17.0	32.4	8.4	29.6	12.6	1.05 (1.00–1.10)
AF	No	15.9	30.7	7.6	28.5	17.3	Ref.
	Yes	18.3	34.9	8.0	27.7	11.2	1.31 (1.25–1.37)
Diabetes	No	16.3	32.6	7.7	28.8	15.3	Ref.
	Yes	17.9	29.3	7.6	28.8	16.5	0.84 (0.80–0.88)
High blood pressure	No	16.2	31.9	7.4	28.0	16.5	Ref.
	Yes	16.9	31.9	7.9	28.4	15.0	1.03 (0.98–1.07)
Smoker	Unknown	26.4	30.8	6.4	24.7	11.7	0.93 (0.86–1.01)
	No	15.9	32.8	8.0	28.3	15.0	Ref.
	Yes	15.8	27.0	6.4	29.4	21.4	0.78 (0.73–0.83)
ADL‐dependent	No	16.0	32.1	7.3	28.1	16.4	Ref.
	Yes	20.1	30.8	10.2	29.0	10.0	0.89 (0.82–0.96)
Living in an institution	No	16.4	31.9	7.4	28.1	16.1	Ref.
	Yes	18.7	31.7	10.2	29.2	10.2	1.26 (1.16–1.37)
Level of consciousness	Alert	15.4	30.6	7.7	29.0	17.4	Ref.
	Drowsy	20.0	37.5	7.8	27.2	7.6	1.40 (1.31–1.49)
	Unconscious	22.4	40.7	8.4	22.5	6.0	1.53 (1.40–1.68)
Stroke subtype	I61	18.3	40.2	7.3	23.2	11.0	1.37 (1.28–1.46)
	I63	16.4	30.8	7.7	28.9	16.2	Ref.
	I64	25.4	24.9	9.5	28.0	12.3	0.73 (0.60–0.88)
Hospital type	University	15.5	32.4	8.0	28.1	16.0	0.92 (0.88–0.96)
	Specialized nonuniversity	17.6	30.8	7.5	28.2	16.0	0.92 (0.87–0.97)
	Community	16.4	33.0	7.8	28.2	14.7	Ref.

Adjusted odds ratios (adj. OR) with 95% confidence intervals (CI) from multiple logistic regression modeling of OAT within 3 hr including all other variables in the table. AF, atrial fibrillation.

### 3.4 Reperfusion therapy

The study included 25,178 patients ≤80 years with ischemic stroke in 2011–2012, of which 2,904 (11.5%) received reperfusion therapy (2,493 thrombolysis, 224 thrombectomy, and 187 thrombolysis and thrombectomy). The reperfusion rate was 15.0% (hospital range: 8.3%–23.4%) in university hospitals, 10.7% (range: 6.1%–15.7%) in specialized nonuniversity hospitals, and 10.5% (range: 2.6%–22.3%) in community hospitals performing thrombolysis.

**Figure 2:**
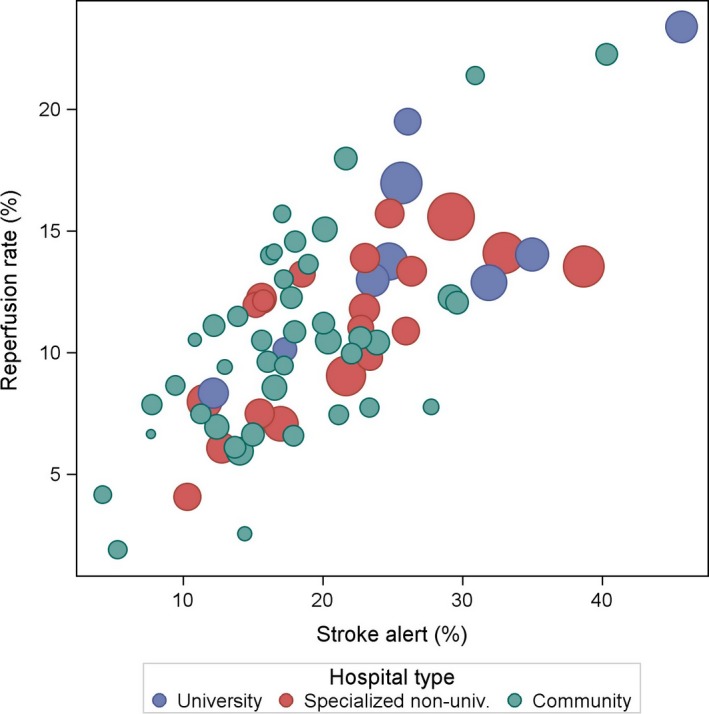
Hospital reperfusion rate (%) in ischemic stroke patients ≤80 years versus hospital stroke alert frequency in all admitted stroke patients, 2011–2012. The size of the bubble corresponds to hospital patient volume

There was a strong correlation between hospital stroke alert frequency and reperfusion rate (*r *=* *.75, *p *<* *.001, Figure 2), but not between hospital reperfusion rate and ambulance service frequency (*r *= −.02, *p *=* *.84).

## 4 Discussion

This study shows substantial between‐hospital variation in the use of stroke alert and ambulance services, also within hospitals of the same type. There are important factors at the individual patient level that are associated with the likelihood of having a stroke alert. Older age, less severe stroke, and living alone are factors that are independently associated with a low chance of having a stroke alert. Even after adjustment for individual patient characteristics, stroke alerts are more common in university hospitals than in nonuniversity hospitals. Hospitals with frequent use of stroke alerts have a high proportion of patients with ischemic stroke being treated with acute reperfusion (thrombolysis or thrombectomy). The use of ambulance services follows a partly different pattern with similar average proportions in university and nonuniversity hospitals, and a predominance of elderly patients with a low level of education. Common to ambulance services and stroke alerts is that they are used more infrequently with immigrants born outside Europe and, as expected, more often with patients with severe stroke.

Most previous studies on stroke alerts have been performed in single centers (Binning et al., 2014; McKinney et al., 2013) or at the regional level (Patel et al., 2011; Prabhakaran et al., 2013), but a very large US study involving 1,585 hospitals has been performed within the Get‐With‐The‐Guidelines‐Stroke (GWTG‐Stroke) project (Lin et al., 2012a, 2012b). Our results agree with those from the US in that there is a marked between‐hospital variation in the use of stroke alerts (Lin et al., 2012b). Our results also agree with previous observations that a high proportion of acute stroke patients with stroke alerts is associated with frequent use of reperfusion therapy such as thrombolysis (Lin et al., 2012a; McKinney et al., 2013; Prabhakaran et al., 2013). Contrary to the US findings (Lin et al., 2012b), we observed that stroke alerts were more common in university than in nonuniversity hospitals.

The use of stroke alerts is low in Sweden compared to the US (23% in this study vs. 64% in US hospitals with >50 stroke admissions per year; Lin et al., 2012b), highlighting a potential to further improve the access to acute reperfusion. To the best of our knowledge, only one previous study has analyzed how patient‐level factors relate to stroke alerts. In the GTWG‐Stroke study, factors associated with lower use of stroke alerts included older age, having diabetes mellitus, and having peripheral vascular disease. Stroke alerts were less likely among black patients than white patients (Lin et al., 2012b). We found socioeconomic factors to be independently associated with the frequency of stroke alerts, and having a low level of education reduced the odds of stroke alert by 5%, being an immigrant of non‐European origin reduced it by 21%, and living alone reduced it by 37%. These estimates were independent of each other. There are probably population subgroups in which socioeconomic factors interact to generate even larger gradients. It is also likely that low awareness of stroke symptoms, language problems, delayed emergency calls, and more patients having passed the upper time limit for treatment when first contacting prehospital health care services have all contributed to the socioeconomic differences in stroke alerts.

Several of our findings on stroke alerts might be explained by more frequent occurrence of obvious contraindications to acute reperfusion in subgroups of stroke patients. For instance, a previous hemorrhagic stroke or a combination of previous ischemic stroke and diabetes are listed as contraindications to thrombolysis by the Swedish Medical Products Agency (Medical Products Agency, 2015). This would help to explain a relatively low frequency in patients with a history of previous stroke, diabetes, and being dependent in ADL before the index stroke or unconscious on arrival at the hospital. Because stroke alerts are associated with shorter time from admission to diagnosis and reversal of anticoagulation in warfarin‐associated intracerebral hemorrhage (Dowlatshahi et al., 2013), it is worth noting that patients who were diagnosed with an intracerebral hemorrhage were more likely than average to have a stroke alert.

At first glance, it would seem paradoxical that ambulance services are used less frequently and stroke alerts more frequently in people with a high level of education compared to those with a low level of education. However, it has previously been reasonably well established that even after adjustment for multiple possible determinants, emergency medical transports are used more often by socioeconomically disadvantaged people (Rucker, Edwards, Burstin, O'Neil, & Brennan, 1997; Svenson, 2000), whereas people with a high level of education have higher awareness of stroke warning symptoms (Hickey et al., 2009).

The present observations help to explain the socioeconomic differences in access to thrombolytic treatment that we have previously described. Compared with stroke patients with primary school education, patients with university‐level education are more often treated with thrombolysis (Stecksen et al., 2014). Our results now show that stroke alerts are more frequent and that OAT times are shorter in patients with a high level of education. The use of ambulance services per se does not seem to follow the same socioeconomic pattern.

Living alone versus cohabiting does not adversely affect the access to ambulance services, but it markedly reduces the frequency of stroke alerts, most likely because of delays in calling 112 (911 in the US) and because of more uncertainties about onset of stroke symptoms. As a result, fewer patients living alone were admitted to hospital within 3 hr of onset of stroke symptoms (present results), and 44% fewer received thrombolytic treatment (Stecksen et al., 2014) compared to patients who were cohabiting. It seems that elderly single people should be especially targeted when campaigns to improve stroke awareness in the population are designed (Desai, Herter, Riccardi, Rorden, & Fridriksson, 2015).

### 4.1 Strengths and limitations

Strength of this study is that it is nationwide and covers all hospitals admitting acute stroke patients in the country and that it has high coverage of all patients admitted to hospital for acute stroke in the country. This study was based on individual‐level socioeconomic information, like the GWTG‐Stroke study (Lin et al., 2012b). Most studies on socioeconomic status (SES) and different aspects of stroke have used SES data based on states, counties, or postal numbers. We acknowledge that geographical‐level SES data may add another dimension of information to the more specific individual‐level SES data; individual‐level and geographical‐regional data should be seen as complementary.

A limitation of this register‐based study is that it was not possible to distinguish between stroke alerts elicited by ambulance services (also called prehospital triage [Prabhakaran et al., 2013] or prehospital notification [Blomberg, Lundstrom, Toss, Gedeborg, & Johansson, 2014]), emergency room prenotification, or other modes of stroke alert. Hospital effects were accounted for in the statistical models, but the associations between patient characteristics and stroke alert frequency remained. This indicates that stroke alerts in themselves, irrespective of how they were applied in each hospital, account for the associations, but it does not exclude that some modes of application might have a stronger relationship than others. Another limitation is that we had no information on negative stroke alerts (i.e., stroke alerts in patients who were subsequently not diagnosed with acute stroke). This includes the relatively small proportion of patients who fully recovered after thrombolysis and were diagnosed with transient ischemic attack. A limitation was also that we did not have information on whether a patient was living in an urban or a rural setting (that could partly explain differences in stroke alerts between different types of hospital).

Results from observational studies like the present one raise the question of possible residual confounding variables that might have influenced some or all of the findings. There are, for instance, many aspects of living conditions that might affect the use of stroke alerts and ambulance services, for example, distance to hospital, that might affect the use of stroke alerts and ambulance services, but these have not been measured. Riksstroke did not include a stroke scale during the study period, and hence we could not specify which stroke signs influenced stroke alerts. There was no information on stroke size or location. Level of consciousness at hospital arrival was used as a surrogate for stroke severity.

## 5 Conclusion

Stroke alerts are low‐technology interventions with a significant potential to improve acute stroke outcome. Because every second stroke alert in Sweden actually leads to reperfusion treatment (Figure 2), it is notable that 50% of stroke patients are living alone in Sweden (Table 1) and that they have a 37% reduction in the likelihood of having a stroke alert. Inequity in other socioeconomic factors, such as level of education and country of birth, also contribute considerably to reduce the use of stroke alerts.

Public stroke warning campaigns have been reported to have varying degrees of success (Mellon, Doyle, Rohde, Williams, & Hickey, 2015; Rasura et al., 2014). The present findings may help to define population groups that should be targeted to make early ambulance calls. Such a specific target group would be elderly people living alone.

## Conflict of Interest

The authors have no conflicts of interest.
