# Supplementary material for: Acute stroke alert activation, emergency service use, and reperfusion therapy in Sweden
Source: Brain Behav. 2017 Mar 15;7(4):e00654. doi: 10.1002/brb3.654 (PMC5390837; doi:10.1002/brb3.654)
Supplement: Supplementary file 1 [file BRB3-7-e00654-s001.docx]

Supplementary Table 1. Stroke alert frequency (%) with 95% confidence intervals (95% CI), 2011–2012. Adjusted odds ratios (Adj. OR) with 95% CI from the multiple logistic regression model including all variables in the table. *) patients who did not use ambulance

| Variable | Category | Valid N | Prop. (95% CI) | Adj. OR (95% CI) | Adj. OR (95% CI)* |
| --- | --- | --- | --- | --- | --- |
| Sex | Women | 23,411 | 20.0 (19.4–20.5) | 0.99 (0.94–1.04) | 0.96 (0.82-1.13) |
|  | Men | 24,955 | 23.3 (22.8–23.9) | Ref. | Ref. |
| Age group | 18–54 | 2,945 | 28.8 (27.2–30.5) | Ref. | Ref. |
|  | 55–64 | 5,448 | 25.8 (24.7–27.0) | 0.94 (0.85–1.05) | 0.72 (0.53-0.96) |
|  | 65–74 | 11,255 | 25.5 (24.7–26.3) | 0.92 (0.83–1.01) | 0.74 (0.56-0.97) |
|  | 75–84 | 15,724 | 21.6 (20.9–22.2) | 0.78 (0.71–0.87) | 0.73 (0.55-0.97) |
|  | 85+ | 12,994 | 15.3 (14.6–15.9) | 0.52 (0.47–0.58) | 0.44 (0.30-0.63) |
| Education | Unknown | 1,343 | 25.2 (22.8–27.5) | 1.03 (0.86–1.24) | 1.19 (0.62-2.26) |
|  | Primary | 22,384 | 19.1 (18.6–19.6) | Ref. | Ref. |
|  | Secondary | 17,166 | 23.2 (22.6–23.9) | 1.08 (1.02–1.14) | 1.03 (0.86-1.23) |
|  | University | 7,473 | 25.3 (24.4–26.3) | 1.01 (0.94–1.08) | 1.18 (0.95-1.47) |
| Country of birth | Missing | 402 | 35.8 (31.1–40.5) | – | – |
|  | Sweden | 41,980 | 21.4 (21.0–21.8) | Ref. | Ref. |
|  | Other Nordic | 2,767 | 21.9 (20.4–23.5) | 1.00 (0.91–1.11) | 1.46 (1.08-1.98) |
|  | Other Europe | 2,141 | 24.6 (22.7–26.4) | 1.02 (0.91–1.14) | 1.25 (0.90-1.73) |
|  | Other | 1,076 | 23.0 (20.5–25.6) | 0.79 (0.68–0.93) | 0.79 (0.49-1.27) |
| Living alone | Missing | 257 | 17.9 (13.2–22.6) | – | – |
|  | No | 23,938 | 26.5 (26.0–27.1) | Ref. | Ref. |
|  | Yes | 24,171 | 17.0 (16.5–17.5) | 0.63 (0.60–0.66) | 0.83 (0.70-0.98) |
| Previous stroke | Missing | 306 | 14.7 (10.7–18.7) | – | – |
|  | No | 36,222 | 22.4 (22.0–22.9) | Ref. | Ref. |
|  | Yes | 11,838 | 19.6 (18.9–20.3) | 0.90 (0.85–0.96) | 0.83 (0.67-1.03) |
| Atrial fibrillation | Missing | 300 | 23.3 (18.5–28.1) | – | – |
|  | No | 34,294 | 21.4 (21.0–21.9) | Ref. | Ref. |
|  | Yes | 13,772 | 22.3 (21.7–23.0) | 1.25 (1.19–1.32) | 1.18 (0.97-1.45) |
| Diabetes | Missing | 163 | 25.8 (19.0–32.6) | – | – |
|  | No | 38,244 | 22.3 (21.9–22.7) | Ref. | Ref. |
|  | Yes | 9,959 | 19.4 (18.6–20.2) | 0.85 (0.80–0.90) | 0.94 (0.77-1.15) |
| Hypertensive medication | Missing | 314 | 24.8 (20.0–29.6) | – | – |
|  | No | 18,623 | 23.3 (22.6–23.9) | Ref. | Ref. |
|  | Yes | 29,429 | 20.7 (20.2–21.2) | 0.96 (0.92–1.01) | 1.06 (0.90-1.25) |
| Smoker | Unknown | 3,783 | 22.6 (21.3–24.0) | 1.01 (0.92–1.11) | 1.16 (0.82-1.64) |
|  | No | 38,450 | 21.6 (21.2–22.0) | Ref. | Ref. |
|  | Yes | 6,133 | 21.9 (20.8–22.9) | 0.87 (0.81–0.94) | 1.08 (0.88-1.34) |
| ADL-dependent | Missing | 978 | 15.8 (13.6–18.1) | – | – |
|  | No | 41,630 | 22.8 (22.4–23.2) | Ref | Ref. |
|  | Yes | 5,758 | 14.9 (14.0–15.8) | 0.70 (0.63–0.77) | 0.78 (0.49-1.25) |
| Living in an institution | Missing | 151 | 19.2 (12.9–25.6) | – | – |
|  | No | 43,424 | 22.3 (21.9–22.7) | Ref. | Ref. |
|  | Yes | 4,791 | 16.6 (15.6–17.7) | 1.13 (1.01–1.25) | 0.70 (0.38-1.27) |
| Level of consciousness | Missing | 551 | 13.6 (10.7–16.5) | – | – |
|  | Alert | 39,436 | 21.0 (20.6–21.4) | Ref. | Ref. |
|  | Drowsy | 5,934 | 27.8 (26.7–29.0) | 1.70 (1.59–1.83) | 2.72 (1.98-3.74) |
|  | Unconscious | 2,445 | 20.1 (18.5–21.7) | 1.02 (0.90–1.15) | 1.22 (0.58-2.60) |
| Stroke subtype | Hemorrhagic | 5,672 | 27.2 (26.0–28.3) | 1.25 (1.17–1.35) | 0.94 (0.70-1.26) |
|  | Ischemic | 42,012 | 21.2 (20.8–21.5) | Ref. | Ref. |
|  | Unspecified | 682 | 10.6 (8.2–12.9) | 0.57 (0.44–0.74) | 0.51 (0.18-1.40) |
| Hospital type | University | 9,893 | 27.5 (26.6–28.4) | 1.58 (1.49-1.69) | 1.36 (1.11-1.67) |
|  | Specialized non-university | 19,242 | 21.7 (21.1–22.2) | 1.27 (1.20-1.34) | 1.12 (0.93-1.32) |
|  | Community | 16,692 | 18.3 (17.7–18.8) | Ref. | Ref. |
